# Age- and Temperature-Dependent Somatic Mutation Accumulation in *Drosophila melanogaster*

**DOI:** 10.1371/journal.pgen.1000950

**Published:** 2010-05-13

**Authors:** Ana Maria Garcia, R. Brent Calder, Martijn E. T. Dollé, Martha Lundell, Pankaj Kapahi, Jan Vijg

**Affiliations:** 1 Department of Biology, University of Texas at San Antonio, San Antonio, Texas, United States of America; 2 Albert Einstein College of Medicine, Department of Genetics, New York, New York, United States of America; 3 National Institute of Public Health and the Environment, Bilthoven, The Netherlands; 4 Buck Institute for Age Research, Novato, California, United States of America; Stanford University Medical Center, United States of America

## Abstract

Using a transgenic mouse model harboring a mutation reporter gene that can be efficiently recovered from genomic DNA, we previously demonstrated that mutations accumulate in aging mice in a tissue-specific manner. Applying a recently developed, similar reporter-based assay in *Drosophila melanogaster*, we now show that the mutation frequency at the lacZ locus in somatic tissue of flies is about three times as high as in mouse tissues, with a much higher fraction of large genome rearrangements. Similar to mice, somatic mutations in the fly also accumulate as a function of age, but they do so much more quickly at higher temperature, a condition which in invertebrates is associated with decreased life span. Most mutations were found to accumulate in the thorax and less in abdomen, suggesting the highly oxidative flight muscles as a possible source of genotoxic stress. These results show that somatic mutation loads in short-lived flies are much more severe than in the much longer-lived mice, with the mutation rate in flies proportional to biological rather than chronological aging.

## Author Summary

DNA mutations are changes in the DNA sequence, including basepair substitutions and genome rearrangements. Mutations are inevitable and are a consequence of errors made in replicating DNA during cell division or repairing damage. Aging is a complex process of functional decline and increased disease risk that has often been ascribed to the accumulation of DNA mutations. In the past we generated a mouse model that allows quantification and characterization of mutations in somatic tissue in a transgene that can be removed and studied in *Escherichia coli*. The results indicated that mutations accumulate with age in a tissue-specific manner. Using a recently generated similar model for *Drosophila melanogaster*, we now show that mutations also accumulate with age in this invertebrate organism. However, the frequency of mutations, already at young age, was found to be much higher than in mice, with a much higher fraction of genome rearrangements. Mutations accumulated much more quickly at higher temperature, a condition which in flies is associated with decreased life span. Hence, we conclude that the burden of DNA mutation is much heavier in short-lived flies than in mammals and increases with age as a function of biological rather than chronological age.

## Introduction

Aging is a complex process with an as yet unclear etiology and a - for most species - poorly described phenotype [1]. Accumulation of somatic mutations, due to imperfect repair and maintenance, has since long been implicated as a universal, major cause of aging [2], [3], but proved difficult to study in higher organisms. Most somatic DNA mutation assays are indirect and based on alterations in phenotypic characteristics, such as the mouse or *Drosophila* spot tests [4], [5]. In the past, we have generated transgenic mouse models harboring chromosomally integrated lacZ-plasmid constructs that can be recovered in E. coli for the subsequent quantification and sequence characterization of a broad range of spontaneous mutations [6]. The results with this system indicate that somatic mutations accumulate in virtually all organs and tissues of the mouse albeit at different rates and varying in the types of mutational events [7].

Very little is known about spontaneous DNA mutation burdens in somatic tissues during aging of different higher organisms. For mammalian species there is evidence that germ line mutation rates in different phylogenetic groups are generally higher in short-lived *Drosophil*a or rodents than in longer lived primates or birds [8]. This has been ascribed to either differences in generation time or selection of increasingly efficient mechanisms of DNA replication and repair during the evolution of long-lived mammals [9]. However, DNA mutation loads in somatic tissues of invertebrates and vertebrates have never been directly compared. We recently generated several lines of *D. melanogaster* harboring a lacZ-plasmid construct identical to the one used to generate the mouse models [10]. This allowed us, for the first time, to directly compare spontaneous somatic mutation frequencies and spectra between a mammal and an insect as a function of age. The results indicate a significantly higher somatic DNA mutation frequency in flies than in mice with especially the fraction of the more toxic genome rearrangements much higher in the former. Like in the mouse, also in flies mutation frequencies increase with age, but at a much higher rate at higher temperature, which correlates with an increased aging rate and shorter life span.

## Results

**Figure 1:**
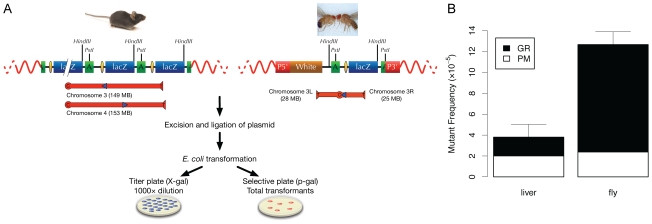
Comparative analysis of somatic mutations in mice and flies. (A) Schematic depiction of the LacZ-plasmid model in *Mus musculus* and *D. melanogaster*. Note that the mouse line 60 contains about 10 head-to-tail organized plasmid copies per integration site, but only two plasmid copies are depicted. For the transgenic flies, each line harbors only 1 copy of the pUR288 plasmid. Individual plasmids can be rescued by excision of genomic DNA with *Hin*d III (H) or *Pst* I (P). After purification from the mouse or fly genomic DNA, self-ligation and transformation into *Escherichia coli C* (Δ*lacZ*, *galE* ^-^) host cells, individual plasmids are recovered in the form of ampicillin-resistant colonies. A small amount of transformants is plated on medium containing X-gal, to determine the total number of plasmids rescued (titer plate). The remainder is plated on media supplemented with the lactose-analogue p-gal, to select only the cells harboring a mutant lacZ (selective plate). The mutant frequency is the ratio of the colonies on the selective plate versus colonies on the titer plate (times the dilution factor). Location and direction of the integration site of the pP[CaSper]vector is shown for line 11. For the mouse, the location and direction of the integrated pUR288 concatamers is shown for line 60. *Lac*Z =  *lacZ* reporter gene; P5′ = 5′ end of (pP[CaSper]); P3′ = 3′ end of (pP[CaSper]); white =  the white selection marker. (B) Direct comparison of spontaneous somatic mutation frequency in mice (3-month old; liver) and flies (1–2-days old; whole body). White bars represent the frequency of point mutations and black bars the frequency of DNA rearrangements. The frequency of all mutations in the fly is greater than that of mouse liver (one-tailed Welch Two Sample t-test, p = 9.04e−05). Error bars are Standard Deviations.

Figure 1A schematically depicts the lacZ-plasmid model for mice and flies. The systems are identical for the two species except for the copy number; while in mice there are approximately 10 copies per integration site, with integration sites on chromosomes 3 and 4, each fly line only contained one copy. Since mutation frequencies are calculated as the number of mutant lacZ copies per total copy number of lacZ-plasmids recovered from a given DNA sample, this has no influence on the mutation frequencies observed. It should also be noted that in general mutation frequencies do not depend on the integration site. While in the past we have observed rare mouse lines with much higher mutation frequencies [11], and there was also at least one fly line that differed significantly from most of the others [10], overall very similar results were obtained for different integration sites. Hence, we felt confident that a direct comparison between the two species would show, for the first time, natural levels of spontaneous mutations in somatic tissue of an invertebrate and a vertebrate.

Figure 1B shows the spontaneous DNA mutation frequencies in the two species, measured as the number of mutant lacZ copies per total number of lacZ copies isolated from mouse liver or whole fly DNA. For mouse liver, mutation frequency is expressed as the average over multiple individual animals and for *Drosophila* as the average over multiple batches of 50 flies. The results indicate a spontaneous mutation frequency in young (1–2 days), male flies of 12.6×10^−5^, which is about 3-fold higher than the mutation frequency in the liver of a young (3 months), male mouse, i.e., 3.8×10^−5^ (Figure 1B). The mutation frequency in the mouse is very similar as what we reported previously and, at young age, does not vary much between tissues [7].

The lacZ-based mutation reporter system allows the characterization of mutations simply by restriction digestion and/or sequence analysis of the plasmids from the E. coli colonies on the selective plates (Figure 1A). In the mouse we found that most mutations are either point mutations (basepair substitutions or small deletions) or genome rearrangements (with one breakpoint in the lacZ gene and the other elsewhere in the mouse genome), with very few intra-lacZ deletions [12]. In the fly we could make the same classification, but while in mouse tissues genome rearrangements were less than 50%, in the fly such events were predominant (Figure 1B). The small fraction of point mutations was dominated by GC to AT transitions and 1-bp deletions (results not shown). Hence, *Drosophila* cells and tissues tolerate not only a 3-fold higher mutation frequency than mouse cells, but also a substantially higher fraction of genome rearrangements.

**Figure 2:**
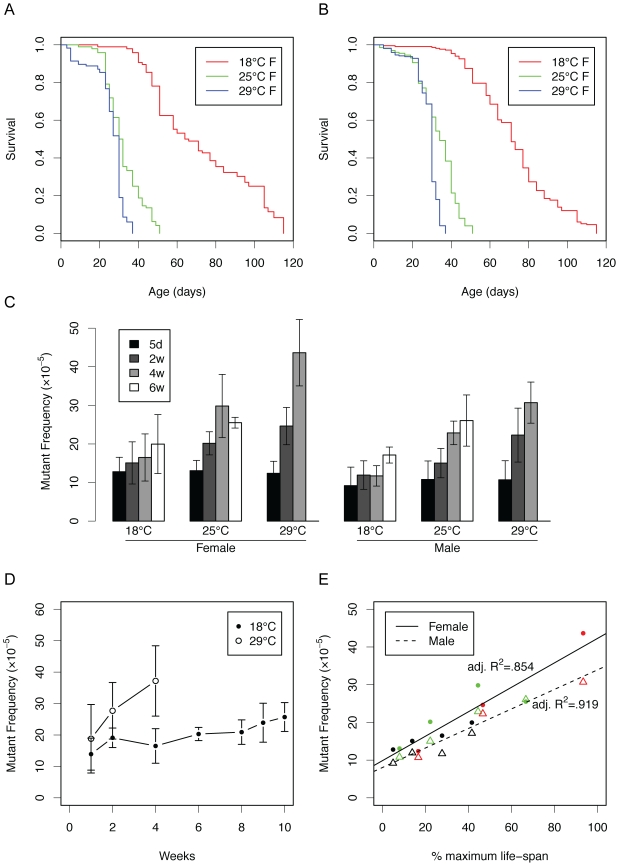
Somatic mutations as a function of life span at different temperatures. (A) Survivalship of female flies grown at 18, 25 and 29°C (p<0.0001 for all comparisons by log-rank test). (B) The same for male flies (p<0.0001 for all comparisons by log-rank test). (C) Mutation frequency in flies maintained at different temperatures with age. Through linear regression analysis, the slopes of all mutation frequencies with chronological time are significant at the p<.01 level except for female flies at 18°C (see Figure S2). The slopes of the different temperatures are significant for females (p = 3.33e−05) and males (p = 4.89e−05). (D) Mutation accumulation at 18 and 29°C in male flies (data are independent of Figure 2C). (E) Mutation frequency of flies as a function of remaining survival time at a given temperature (data are from Figure 2A–2C). Black = 18°C, green = 25°C, red = 29°C, circle = female, triangle = male. Error bars are standard deviations.

Mutations are generally assumed to be associated with cell division when they arise, for example, as errors in repairing damage to DNA. *Drosophila* is a postmitotic organism and contains very little cell proliferative activity as adults. Hence, one would predict that in contrast to the mouse, *Drosophila* would not accumulate mutations during its adult life. Lifespan in *Drosophila* is temperature-dependent and longevity decreases exponentially with increasing temperature between 12 and 30°C [13]. This may be caused by an increase in the rate of metabolic processes, presumably speeding up the aging process through the accumulation of damage. As shown in Figure 2A and 2B, life span of both female and male lacZ transgenic flies is indeed temperature-dependent. While at 29°C the flies live very short with no survivors after 30 days, at 18°C they can live three times as long.

Mutation frequencies at the lacZ locus appeared to be both age and temperature-dependent (Figure 2C). Indeed, this is very clear from Figure 2D where we plotted side by side the age-related increase of lacZ mutation frequencies in male lacZ flies at 18 and 29°C. Of note, these results were obtained in an experiment independent from the experiment that gave rise to the data presented in Figure 2C. Similar results were obtained when using another lacZ fly line, line 5 with the lacZ reporter integrated elsewhere on the same chromosome (Figure S1; see also ref. [10]).

From Figure 2D it is obvious that the rate of the age-related increase differs dramatically between 18 and 29°C. Indeed, when plotting the mutation frequencies in Figure 2C as a function of the remaining life span, as obtained from the survival curves in Figure 2A and 2B, highly significant correlations were observed both for males and females (Figure 2E). This suggests that mutations accumulate as a function of biological rather than chronological age. If true, this would predict that when for each temperature mutation frequencies are plotted against chronological rather than biological time they would be statistically significantly different from each other. As we show in Figure S2, this was indeed the case, for both line 11 and line 5.

**Figure 3:**
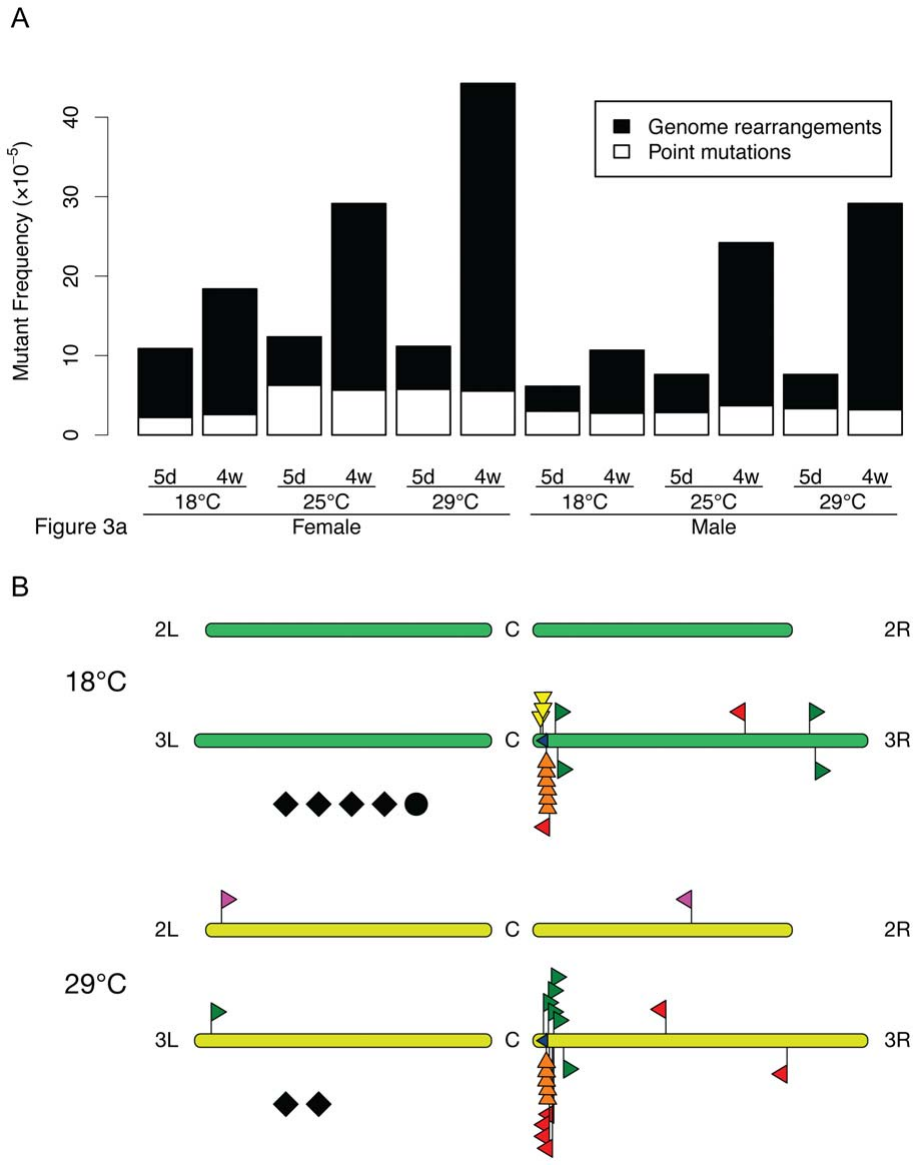
LacZ mutation spectra in *D. melanogaster* at different temperatures. (A) Frequencies of point mutations (white) and DNA rearrangements (black). Each determination point is based on 60 mutants, taken from the plates used to generate the data in Figure 2C. The accumulation of genome rearrangements was statistically significant (p = 0.0043 in males and p = 0.0260 in females). No significant accumulation of point mutations was detected. (B) Physical maps of the second breakpoint for the genome rearrangements in the fly at 18 and 29°C.

Next, we considered the possibility that increasing the temperature could alter the type of mutations. Hence, we compared the mutation spectrum in flies of 4 weeks old at 18 and 29°C. The results show that in both cases the far majority of the mutations were genome rearrangements, the accumulation of which with age and temperature was statistically significant (Figure 3A). The point mutation loads are fairly constant, but their numbers were too small to draw the conclusion that only genome rearrangements accumulate. However, it is clear that the temperature does not dramatically affect the mutation spectrum as we have seen, for example, after treatment of the flies with the known point mutagen ethyl nitrosourea [10]. Next, as we have done previously for the mouse [14], we physically characterized the rearrangements to assess if the mechanism by which these events arise might be different between the two extreme temperatures (Table S1).

Like in the mouse, in flies we observed both intra-chromosomal and extra-chromosomal events (Figure 3B). The latter were defined as translocations of which there were very few in the fly. In the fly, virtually all intra-chromosomal events were either internal lacZ deletions or events that involved a breakpoint close to the integration site of the reporter gene. The latter could be subdivided, based on the direction of the sequence that was recovered from the mutant plasmid and mapped to chromosome 3, as deletions, inversions or more complex intra-chromosomal recombinations, which included transpositions (not to be confused with the P element transpositions causing hybrid dysgenesis in *Drosophila*) and complex, undefined events. While the types of genome rearrangements observed were similar to what we previously reported for the mouse, the number of rearrangements close to the integration site was much higher for the fly. In mouse tissues we found many more very large events, including many translocations [14]. This is to be expected because *Drosophila* has less and much shorter chromosomes than the mouse and therefore less opportunity for accommodating extra-chromosomal recombinations or very large intra-chromosomal events.

As shown in Figure 3B, no significant differences between 18 and 29°C were observed. Of note, at neither temperature we observed obvious sequence homologies at the break points. Hence, most likely these mutational events are a consequence of erroneous non-homologous end joining of DNA double-strand damage. These results indicate that increasing temperature merely accelerates mutation accumulation without significantly altering the spectrum. This would be expected when temperature affects the basic rate of aging.

**Figure 4:**
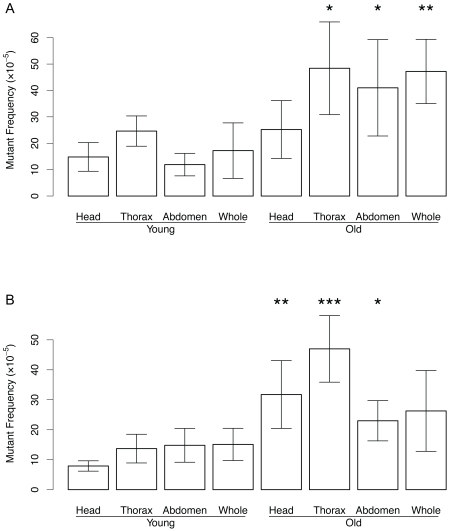
Mutation frequency in DNA from the head, thorax, and abdomen. Of young and old, female (A) and male (B) flies maintained at 29°C. *p<0.05; **p<0.01, ***p<1e−5 (t-test of old vs. young). Mutation frequency of old male thorax was significantly different from old male abdomen (p = 9.13e−05) and old male head (p = 0.01092). Mutation frequency of old female thorax was different only from old head p = 0.03318. Error bars are Standard Deviations.

The similar mutation spectrum between flies aged at the two extreme temperatures strongly suggests that similar types of DNA damage drive the generation of these mutations. Since our mutation frequency determinations were based on whole flies we were not able to tell where in the fly the mutations were generated. Mutations are often considered to arise as errors during DNA replication. While *Drosophila* is generally considered a postmitotic organism, mitotically active cells are present in the abdomen, i.e., the gonads and the gut. Hence, we analyzed mutation frequencies at the lacZ locus in abdomen, thorax and heads of young (1 week) and old (3 weeks), female (Figure 4A) and male (Figure 4B) flies at 29°C. Somewhat surprisingly, the results indicate that most age-related mutations had accumulated in the thorax, not the abdomen. The higher mutation frequency in thorax as compared to abdomen of old flies was statistically significant for males, but not for females; females merely showed a higher mutation frequency in thorax as compared to the heads (also true for males). No significant differences in mutation spectrum were found between body parts; virtually all mutations were genome rearrangements (not shown).

## Discussion

Somatic DNA mutations are random and almost always have adverse effects or are neutral. In cancer, random mutations are selected by providing the host cell with attributes allowing it to escape normal growth constraints, invade tissues and evade host defense systems, such as the immune response. However, increasing loads of random mutations also adversely affect cellular fitness, as has been demonstrated conclusively in E. coli [15]. In higher organisms, such as mice and flies, somatic mutations may exert adverse effects mainly by deregulating gene control. Hence, one would expect somatic mutation frequencies to reach levels that are still compatible with life without compromising reproduction at early age.

Using a similar reporter system for *Drosophila* as we previously used in mice, we now show significantly higher and potentially more severe DNA mutation loads at the lacZ locus in tissues of the fly as compared to the mouse. While the lacZ reporter is not expressed, acting as a neutral target gene, mutation detection requires inactivation or partial inactivation of the lacZ-encoded β-galactosidase activity [16]. Therefore, our present data underestimate the total somatic mutation rate in the organism.

Germ line mutation rates, which can now be derived from direct sequence comparison between individual animals [17], can vary between 1 and 10×10^−6^ per average gene per generation [9], [18]. However, somatic mutation rate is not subject to the same selection constraints as germ line mutation rate. Our current data indicate that the frequency of somatic mutations per locus in flies is 3-fold that of a mammal (Figure 1B). While the fly has a smaller genome than the mouse (about 16-fold), it is more compact in the sense that its gene density is much higher. Hence, random mutations should be more likely to have an adverse effect on the fly genome than on that of the mouse. Moreover, the fly shows a much higher proportion of genome rearrangements - mutational events that generally have a much higher functional impact than point mutations - than the mouse.

Our results in *Drosophila* also indicate, for the first time, that the rate of somatic mutagenesis is a function of both age and temperature, with temperature as the main factor. Since increased temperature reduces life span of flies, it is tempting to speculate that somatic mutation rate is causally related to the rate of aging. For the mouse this is supported by our previous results indicating reduced spontaneous mutation frequencies in mice subjected to caloric restriction [19], an intervention leading to extended life span [20]. We did not find a similar result for the fly, however [21], which is in keeping with results by Mair et al., showing that DR extends life span in *Drosophila* entirely by reducing the short-term risk of death [22]. This difference in response to DR between mice and flies may reflect a difference in the modes of dietary restriction, which is inherently more complicated in flies than in rodents [23].

Interestingly, Mair et al. [22] also demonstrated that reduced temperature, in contrast to the effect of DR, may well increase the life span of flies by reducing the accumulation of aging-related damage. While we have no direct evidence regarding the source of somatic mutations in *Drosophila*, elevated temperatures increase respiration rate in ectotherm animals and thus the production of mitochondrial reactive oxygen species (ROS) [24]. ROS is considered as a major cause of aging [25] and produces a multiplicity of alterations in DNA, including chromosomal aberrations, probably as a consequence of DNA double-strand breaks [26]. Treatment of the lacZ *Drosophila* lines with paraquat, a widely used herbicide that produces ROS in cells, resulted in a significant elevation of somatic mutations [21], most or all of which were genome rearrangements (results not shown). Hence, it is conceivable that the genome rearrangements observed in flies are a result of increasing amounts of ROS generated as a function of the temperature. This would explain why the amount but not the type of mutation depends on both age and temperature.

Our results indicate that while mutations are generally attributed to errors during replication and thus require cellular proliferation most of the mutations in *Drosophila* accumulate in the thorax, a postmitotic organ (Figure 4). In this respect we speculate that high levels of oxidative stress are generated by the flight muscles, which have a very high metabolic rate. This would induce DNA double-strand breaks. Subsequent errors during the repair of the damage, for example, mis-annealing of different DNA ends by non-homologous end-joining or errors during homologous recombination, using the homologous chromosome or sequences elsewhere on the same chromosome as exchange partner rather than its sister chromatid [27]. Interestingly, after crossing the lacZ flies with a line harboring a defect in the BLM gene (DmBlm) [28] - which is required for accurate repair of DNA double-strand gaps by homologous recombination - the age-related accumulation of genome rearrangements was significantly elevated. The DmBlm mice lived significantly shorter than the wildtype control animals (Garcia, A., et al., submitted). Hence, genome rearrangements can evidently accumulate during adult life, possibly as a consequence of errors during DNA double-strand break repair. This is not dependent on cell proliferation. As we demonstrated previously in cultured mouse cells, large rearrangements are easily induced in non-dividing cells by hydrogen peroxide, an oxidative agent [29].

The tolerance of *Drosophila* for severe genomic mutation loads is remarkable and may reflect a reduced level of gene regulatory intricacy as compared to mammals [30]. In the mouse, sizable fractions of genome rearrangements could disrupt the many long-distance gene regulatory interactions and might be unsustainable. The evolution of more complex species with longer life spans and more numerous cell divisions has been associated with more complex mechanisms for gene regulation, which most likely required increasingly sophisticated systems for replication and repair to prevent the deleterious effects of genome rearrangements.

## Materials and Methods

### Mice

Male lacZ mice of line 60 were used with integration sites on chromosomes 3 and 4 at approximately 10 copies per integration site [31]. They were maintained in the animal facilities of the University of Texas Health Science Center at San Antonio on a 14-h light/10-h dark cycle at a standard temperature of 23°C. Standard lab chow (Harlan Teklad, Madison WI) and water were supplied ad libitum. Animals were sacrificed by cervical dislocation following CO_2_ inhalation. Ethical approval to carry out this work on animals was provided by the IACUC of the University of Texas Health Science Center.

### *Drosophila* stocks and maintenance

The transgenic lines 11 and 5, harboring the lacZ reporter gene on chromosome 3R, was used in this study and generated by P-element transformation using a w1118 background [10]. Flies were raised on standard cornmeal-molasses-agar-yeast medium with propionic acid added to the food as an anti-fungal agent. All mating, egg laying and hatching were done at 25°C and 60% humidity. Once hatched, one to two day old flies were transferred to cages containing 200–300 flies and maintained at 18°C, 25°C and 29°C, respectively, throughout the experiment. Males and females were maintained together in the same cage throughout the experiment but they were separated by sex during collection time. Food was changed every other day and samples of 50 flies for each sex were collected at 5 days, 2 weeks, 4 weeks and 6 weeks. For the longevity study of line 11 at 18°C additional male samples were also collected at 8, 9 and 10 weeks. Samples were stored at −80°C. For the body parts study, flies were obtained as described above and 1–2 days old flies were transferred to a 29°C incubator. Samples of 100 flies for each sex were collected at 1 week (young) and 3 weeks (old) of age and stored at −80°C. The dissection was done under the microscope (Stemi SV 6 Zeiss) in the presence of dry ice. Flies were dissected into head, thorax and abdomen and mutant frequency determinations were carried out for each sex and age group.

### Survival determination

For the survival determination, fly deaths were recorded every 2–3 days and dead flies were removed from the cages. The total number recorded disregards rare escape or accidental death of flies. All life span determinations were performed independently of the mutation frequency assessments.

### Genomic DNA isolation

Each sample consisted of 50 pooled male or female flies. Flies were homogenized in 600 µl of lysis buffer (10 mM Tris-HCl, pH 8.0; 10 mM EDTA; 150 mM NaCl) in 2 ml eppendorf tubes using a battery-operated pestle. To the homogenate, 12 µl of Proteinase K (25 mg/ml), 60 µl 10% SDS and 10 µl RNAse A (20 µg/ml) were added and samples were incubated at 65°C while rotating during 30 min. Genomic DNA was subsequently extracted from these samples using phenol/chloroform.

### Mutation analysis

The mutation frequency was determined as described in detail elsewhere [31]. Briefly, isolated DNA (either from mouse liver tissue or from 50 flies was digested for one hour at 37°C with Hind III (40 U) in the presence of magnetic beads coated with lacI-lacZ fusion protein. The lacZ plasmid was then eluted from the beads by incubation with IPTG, circularized by ligation with T4 DNA ligase (Biolabs), precipitated with ethanol and used to electrotransform E. coli (ΔlacZ, galE -). Each mutant frequency determination point was based on at least 3 replicates of the same sample, i.e., three 50-fly groups from the same population, with a minimum of 100,000 colonies for each rescue. The mutation frequency is the ratio of colonies growing on the selective plate vs. the total number of recovered plasmids from the DNA sample (as measured on the titer plate). Hence, mutation frequencies as determined with this system reflect a ratio and do not depend on the amount of DNA. They are expressed on a per locus basis as the number of mutant lacZ copies for a given number of lacZ copies isolated from the in vivo situation. LacZ plasmids from mutant colonies were further characterized as described in detail elsewhere [31]. Sequence reactions of purified mutant plasmids were outsourced to Sequetech corporation (Mountain View, CA). The returned chromatograms were analyzed with Sequencher (Gene Codes, Ann Arbor, MI). Analysis of large rearrangements consisted of non-lacZ sequences were carried out using the fly genome database (http://www.flybase.org). After alignment with the *D. melanogaster* sequence, the chromosomal origin of the flanking sequences was determined and the orientation and the type of chromosomal rearrangements deduced as described [14].

### Statistical analysis

Statistical tests were performed using R (http://www.r-project.org). Linear regressions were used to model mutation frequency as a function of chronological and biological age. Differences in mutation frequency between body parts were evaluated using the Welch two-sample t-test.

## Supporting Information

Figure S1 Plot of mutation frequency versus chronological age for female line 5 flies at 18° and 29°C.
(0.01 MB PDF)

Figure S2 Plots of mutation frequency (×10^−5^) versus chronological age at 18, 25, and 29°C for male and female flies of lines 5 and 11. Slopes are significantly different from 0 at the p<0.01 level for all slopes except for female line 5 @ 18°, male line 5 (18, 25, 29°), and female line 11 @ 18°C. The slope for male line 5 is significantly different from 0 at the p<0.05 level for 25° and 29°C.
(0.24 MB PDF)

Table S1 Somatic genome rearrangements in *D. melanogaster* at 18°C and 29°C.
(0.03 MB XLS)
