# Supplementary figures and images for: Age- and Temperature-Dependent Somatic Mutation Accumulation in Drosophila melanogaster
Source: PLoS Genet. 2010 May 13;6(5):e1000950. doi: 10.1371/journal.pgen.1000950 (PMC2869313; doi:10.1371/journal.pgen.1000950)

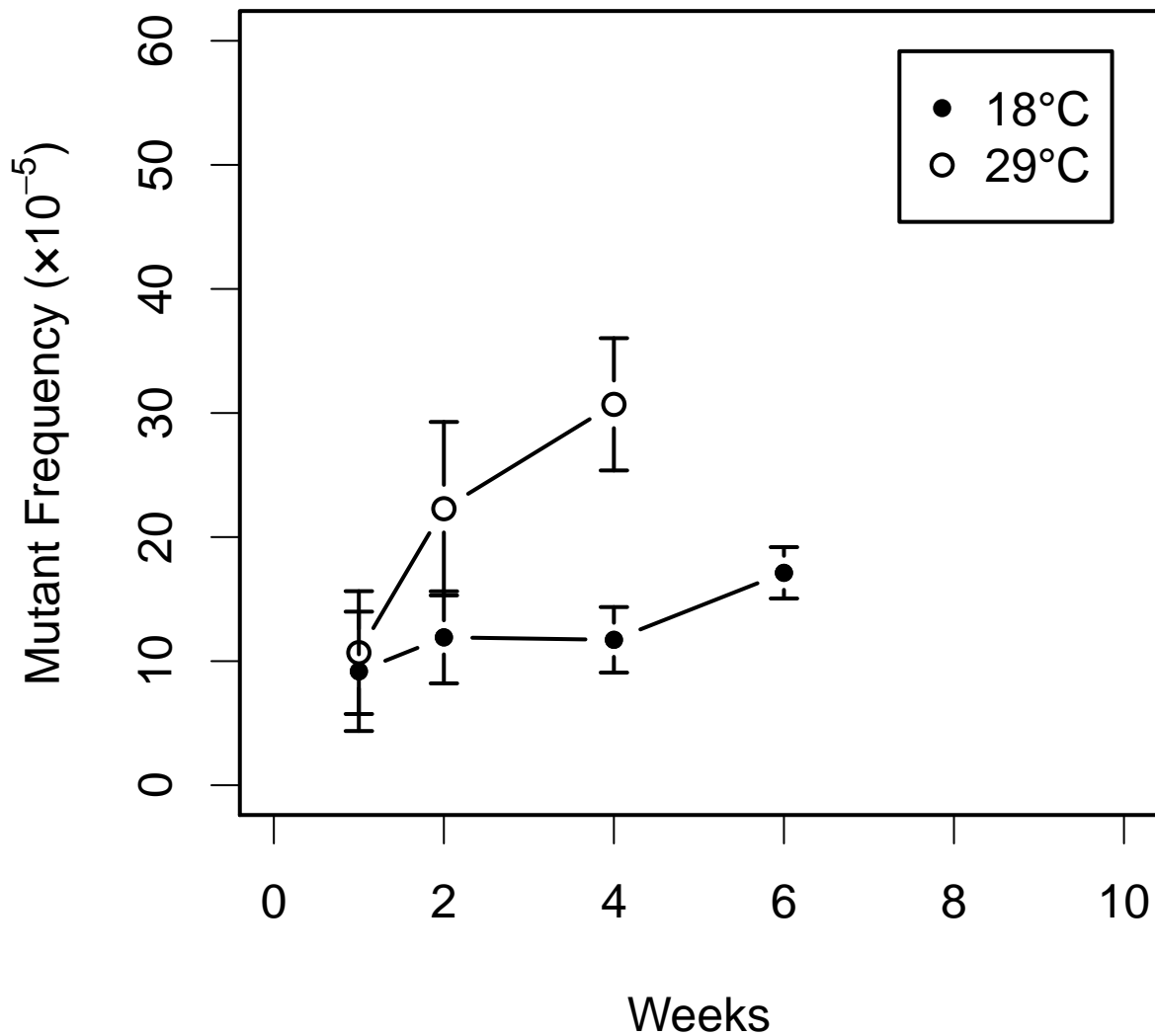

Supplement: Figure S1 — Plot of mutation frequency versus chronological age for female line 5 flies at 18° and 29°C. (0.01 MB PDF) [file pgen.1000950.s001.pdf]

female line 5

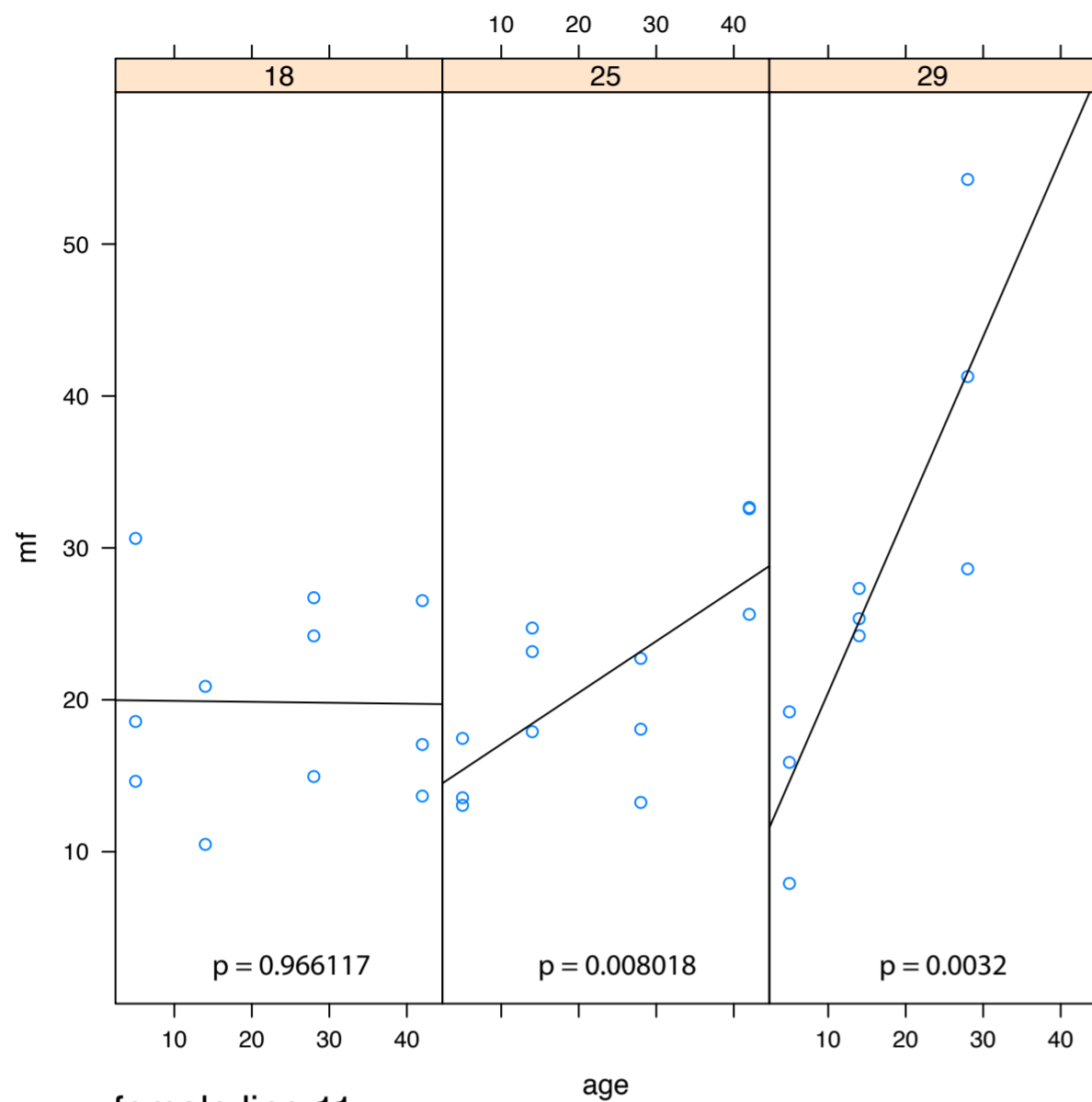

male line 5

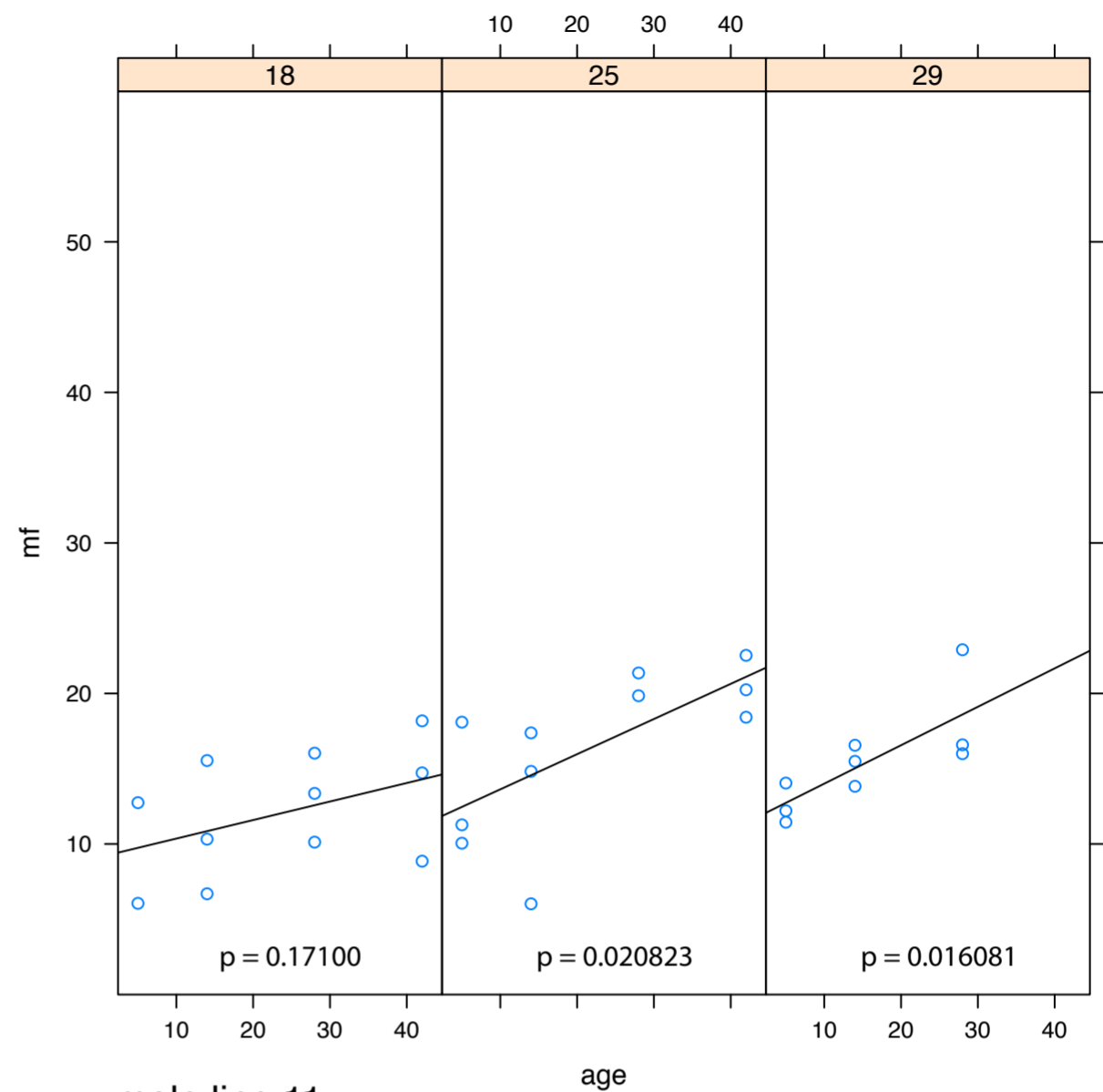

female line 11

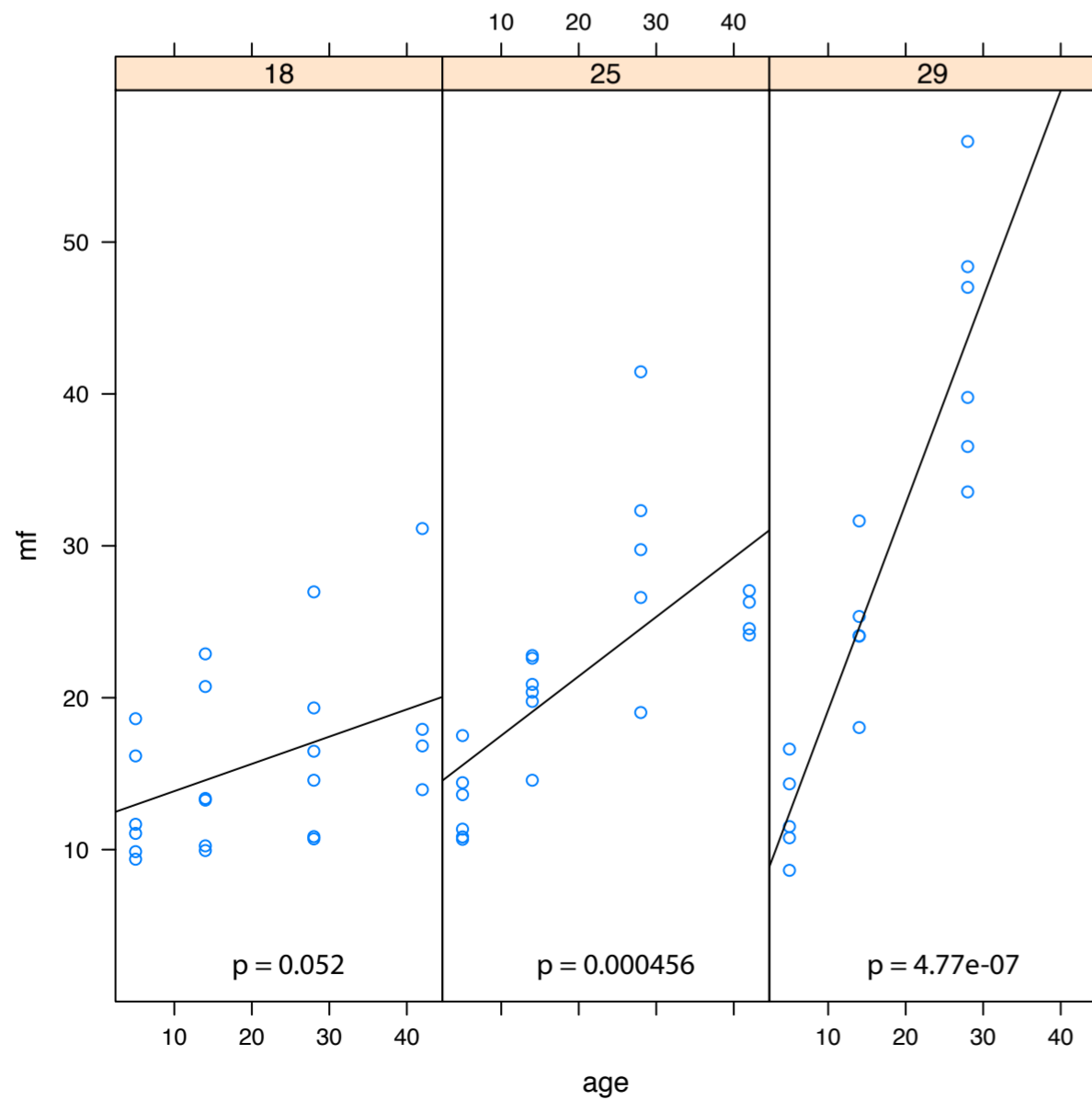

male line 11

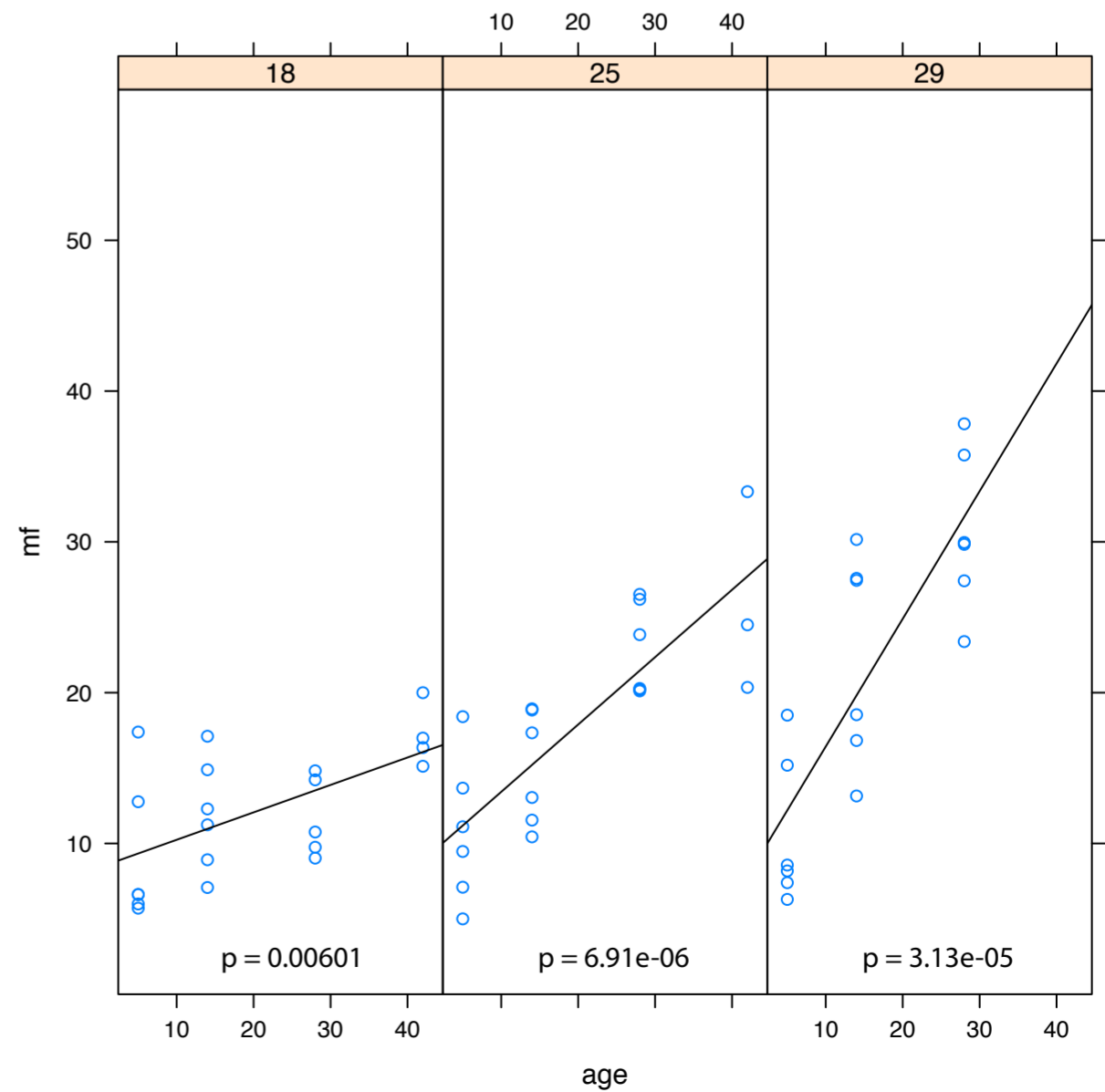

Supplement: Figure S2 — Plots of mutation frequency (×10−5) versus chronological age at 18, 25, and 29°C for male and female flies of lines 5 and 11. Slopes are significantly different from 0 at the p<0.01 level for all slopes except for female line 5 @ 18°, male line 5 (18, 25, 29°), and female line 11 @ 18°C. The slope for male line 5 is significantly different from 0 at the p<0.05 level for 25° and 29°C. (0.24 MB PDF) [file pgen.1000950.s002.pdf]
